# Laminin Immunostaining in Biopsies as a Useful Biomarker of Early Invasion in Actinic Cheilitis and Differential Diagnosis Between Actinic Cheilitis and Lip Cancer: New Insights

**DOI:** 10.1007/s12105-022-01504-y

**Published:** 2022-10-27

**Authors:** D. Vageli, P. G. Doukas, K. Zacharouli, V. Kakanis, M. Strataki, A. Zioga, C. Skoulakis, G. Koukoulis, M. Ioannou

**Affiliations:** 1 Department of Pathology, Faculty of Medicine, School of Health Sciences, University of Thessaly, Biopolis, 41500 Larissa, Greece; 2 Department of Surgery, Yale Larynx Laboratory, Yale School of Medicine, New Haven, CT 06510 USA; 3 Department of Neurology & Sensory Organs, Faculty of Medicine, School of Health Sciences, University of Thessaly, Biopolis, 41500 Larissa, Greece

**Keywords:** Actinic cheilitis, Epithelial dysplasia, Lip cancer, Laminin, Immunohistochemistry, Basement membrane

## Abstract

**Background:**

Squamous cell carcinoma of the lip (LSCC) and oral cavity can be life-threatening if not diagnosed early. Precancerous lesions like actinic cheilitis (AC), can transform into LSCC. Laminin is a fundamental component for basement membrane (BM) and its integrity may prevent neoplastic invasion. Therefore, laminin immunostaining of BM may be useful in identifying early invasion in actinic cheilitis and thus in the differential diagnosis between AC and invasive LSCC or high-grade epithelial dysplasia (ED).

**Materials and methods:**

Biopsies from 46 patients with oral lesions were histologically analyzed and immunohistochemically stained for laminin-1.

**Results:**

AC was diagnosed in 34 patients and LSCC in 12 patients, including 3 patients with AC and concomitant high-grade ED/in situ carcinoma. Laminin-1 immunostaining revealed intense and linear expression of the BM in AC with low-grade ED. Loss of laminin expression was observed in LSCC. Intracellular laminin expression in parabasal cells was noted in AC with high-grade ED/in situ carcinoma.

**Conclusion:**

Laminin immunostaining could be useful in identifying AC cases suspected of early invasion. It could also contribute to the histopathological differential diagnosis between AC with low- and high-grade ED and between AC and invasive LSCC. The findings of this study provide new insights into the mechanism involved in the progression process of AC into LSCC, encouraging preclinical studies that may document the stochastic role of laminin in this process.

## Introduction

Lip and oral cavity cancers represent the 16th overall and the 15th deadliest cancer worldwide with an increasing incidence in the last decades [1–3]. It is estimated that in 2022 the number of lip and oral cavity cancer-related deaths worldwide will be 80,736 [2]. Similar to other head and neck cancer subtypes, the majority of cancers of the lip and oral cavity are squamous cell carcinomas (SCC) (> 90%) which are characterized by a high degree of local invasion and high rate of metastasis that directly affects the patient prognosis [4–8]. The early diagnosis of lip and oral cavity cancer and premalignant lesions is essential [9]. In particular, lip squamous cell carcinoma (LSCC) can be prevented either by reducing exposure to solar ultraviolet (UV) radiation, which is its most common cause [10], or by screening for precancerous lesions [2]. Precancerous lesions of the lip and the oral cavity represent mucosal lesions, such as oral leukoplakia, oral erythroplakia, proliferative verrucous leukoplakia, oral submucous fibrosis, or actinic cheilitis (AC), while the latter may progress to SCC of the lip (LSCC) [11] depending on specific prognostic factors such as epithelial dysplasia (ED) [12–14].

AC, also known as actinic cheilosis or solar cheilitis, is the most common precancerous lesion of lip and oral cavity cancers. AC was first described by Ayres in 1923 [15, 16] as a chronic inflammatory process that affects the lower lip in 95% of cases and is most commonly caused by chronic exposure to sunlight or artificial ultraviolet radiation [17]. Its clinical presentation includes dryness, erythema, and atrophy at the edge of the vermilion border of the lower lip in predominantly white middle-aged males [18]. AC can progress into invasive LSCC [17–21] with an estimated incidence ranging from 1.4 to 36% over one to thirty year intervals. Some common risk factors associated with malignant transformation of AC are tobacco smoking, alcohol abuse, HPV, race, family, genetic predisposition, immunosuppressive status, poor diet, and socioeconomic factors [21]. Histopathological features of AC include hyperplasia, acanthosis or atrophy of the squamous stratified epithelium, hyperkeratosis, and/or different degrees of ED. Also, in subjacent connective tissue, basophilic degeneration of collagen fibers called solar elastosis is usually detected [22].

According to Dancyger et al., AC pathogenesis is similar to cutaneous actinic keratosis or solar keratosis [23]. In particular, several genes have been implicated in the progression of AC into LSCC, including tumor suppressor gene *p53*, anti-apoptotic *BCL2*, *Ki-67*, and Murine Double Minute 2 (*MDM2*) tumor-suppressor protein [24]. Although there is a high incidence of UV-specific *p53* mutations in both AC (80%) and SCC (90%) [25], p53 protein immunoreactivity has not been considered a marker of the malignant transformation of AC into SCC [26]. On the other hand, the majority of precancerous lesions of the lip, including AC, may not always progress into invasive cancer. Therefore, it is necessary to establish reliable diagnostic histopathologic markers to distinguish precancerous from cancerous lesions and then apply them in clinical practice in order to detect early malignant transformation of the epithelium [27].

Laminin is the most abundant non-collagenous component of all basement membranes [28]. Laminins are a family of heterotrimeric glycoproteins composed of three different gene products, α, β, and γ chains. These chains are assembled into a cross-shaped heterotrimer αβγ^3^ [28, 29]. There are at least 16 different laminin isoforms named based on the chain composition [29]. The α1 chain together with the β1 and γ1 chains forms the prototypic EHS laminin (laminin-1) and is expressed in different basement membranes [30]. Laminins are involved in several biological processes such as cell differentiation, cell adhesion, migration, proteolytic activity, cell proliferation, and metastatic growth [29–32]. Under the light microscope, laminin presents a continuous expression in the normal oral mucosa or oral hyperplastic lesions [33, 34]. On the contrary, previous studies suggest a discontinuous distribution of laminin from epithelial hyperplasia to epithelial dysplasia [35–38]. Cytoplasmic laminin expression levels have also been found to be higher in poorly differentiated aggressive oral SCC [32, 35].

The discontinuous pattern of laminin expression in the basement membrane (BM) as well as the absence of its expression might be useful in the early diagnosis of precancerous lesions of the lips as well as in predicting the biological progression toward malignancy. Therefore, the contribution of laminin to the transformation of AC into LSCC needs further clarification. Here we hypothesized that laminin expression levels are altered during lip carcinogenesis. We also hypothesized that immunohistochemical (IHC) expression of laminin might be a useful diagnostic marker to identify early invasion and distinguish cancerous from precancerous lesions. To investigate our hypothesis, we analyzed laminin expression by IHC analysis in biopsies taken from patients with AC of low-grade and high-grade ED and correlated them with its expression in LSCC biopsies. In order to investigate the possible utility of this marker in the differential diagnosis of precancerous lesions, we examined the integrity of the BM as well as cytoplasmic staining patterns. Understanding the mechanism involved in the progression process of actinic cheilitis into LSCC may provide useful histopathologic markers for its early detection, as well as targets for its therapeutic efficacy to be evaluated by further investigation in larger studies.

## Materials and Methods

### Tissue Samples

Lip tissue samples were represented by surgical biopsies from 46 patients, 37 males, and 9 females; (mean age of 67 years). These biopsies were performed for diagnostic purposes. The biopsies had been routinely fixed in 10% buffered formalin solution and then processed and embedded in paraffin blocks (FFPE). All biopsies were retrieved from the archived files of the Pathology department at the University of Thessaly, Greece. [Ethical approval (#28,255/06-07-22) was obtained by the local Ethics Committee of the University of Thessaly]. All FFPEs used in this study were documented with unique de-identified identification numbers.

## Histopathologic Evaluation

Histologic evaluation was performed by microscopic examination of 3–4 μm tissue sections stained with hematoxylin and eosin (H&E). ED was classified as low or high grade according to the diagnostic criteria provided by World Health Organization (WHO) [38]. According to WHO, ED was classified as low-grade (mild) or high-grade (moderate to severe), based on architectural and cytological criteria. These criteria include changes such as irregular stratification, loss of cellular cohesion, drop-shaped rete ridges, increased nuclear-cytoplasmic ratio, hyperchromatism, nuclear and cellular pleomorphism, typical and atypical mitoses, increased size, the number of nucleoli, dyskeratosis, and keratin pearls. In particular, ED was characterized as low-grade (mild dysplasia) when the cellular atypia alterations were restricted to the basal and parabasal region of the epithelium, not exceeding the inferior third. ED was characterized as high-grade when alterations were extended from the basal layer to the middle-third of the epithelium (moderate dysplasia) or a level above the midpoint, affecting more than two-thirds of the epithelium (severe dysplasia or in situ carcinoma) [39].

## Immunohistochemistry

IHC analysis was performed to detect laminin-1, since its expression is largely limited to the epithelial BM [40]. Following previously established protocols [41, 42], 3–4 µm tissue sections were cut from FFPEs using a Leica TP1020 microtome and dried overnight at 60 ℃. After deparaffinization in xylene, the sections were rehydrated in decreasing ethanol solutions. Antigen retrieval was achieved by treating tissue sections with proteinase K for 10 minutes at 37 ℃. After antigen retrieval, the sections were washed in phosphate-buffered saline (PBS). Then they were incubated in 0.3% hydrogen peroxide for 10 minutes to block endogenous peroxidase. Immunostaining was performed with the primary antibody (Laminin, clone LAM89, isotype IgG1, Thermo Scientific, dilution 1:50) for 30 min. A polymer detection system (Genemed Biotechnologies) was then added followed by incubation for 30 min. Bound antibodies were visualized by using 0.05% 3,3′-diaminobenzidine solution (DAB, DAKO). Finally, sections were counterstained with 1% hematoxylin and mounted in DPX mounting medium (BDH Laboratory Supplies, UK). The expression of laminin in the BM of normal surgical margins was considered an internal positive control. Negative controls were performed by omission of the primary antibody and substituting the antibodies with non-immune sera.

## Results

### Actinic Cheilitis Occurs with Concomitant Low- to High-grade Dysplastic Lesions and/or Invasive Squamous Cell Carcinoma

**Fig. 1:**
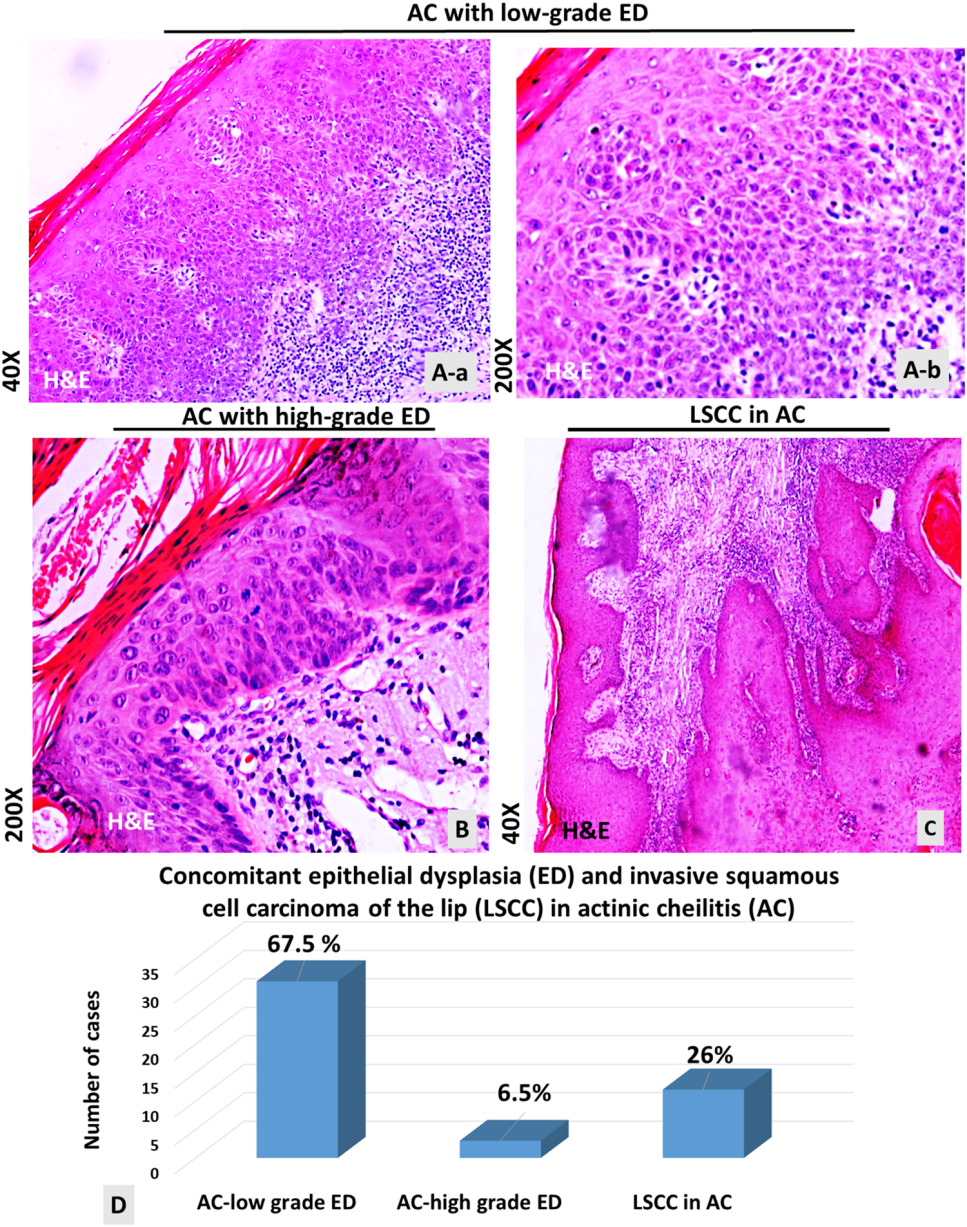
Actinic cheilitis (AC with concomitant low-grade to high-grade epithelial dysplastic (ED) lesions or invasive squamous cell carcinoma of the lip (LSCC). Hematoxylin and eosin (H&E) histologic staining. **A **Actinic cheilitis with low-grade ED (a) Original magnification x40. (b) Original magnification of 200x. **B** Actinic cheilitis with-high grade ED (original magnification x200). **C** Invasive LSCC developed in AC (original magnification of x40). **D** Percentages of cases developed ED and/or LSCC in AC

Microscopic examination of lip tissue sections revealed 34 cases of AC and 12 cases of LSCC. Specifically, microscopic examination of tissue sections stained with H&E revealed 31 AC cases with low-grade dysplasia (Fig. 1A), 3 AC cases with high-grade dysplasia (Fig. 1B), and 12 SCC cases developed in AC (Fig. 1C). Figure 1D shows the percentage of cases that developed ED and/or LSCC in AC.

All specimens that showed the histopathological features of AC were characterized by large hyperchromatic nuclear, abnormal cell shape, abnormal mitosis in parabasal layers, loss of basal cell polarity, a disorder of squamous epithelium stratification, dyskeratosis, and bulbous form shaped rete pegs (Fig. 1A, B). Chronic inflammatory infiltration was also observed. Epithelial atrophy was observed in nine cases (Fig. 1B). The SCC of our study showed neoplastic infiltration into the dermis (Fig. 1C). These cases were developed from AC, the features of which were evident in other epithelial foci.

### Laminin Staining of the Basement Membrane is Discontinued in Biopsies with High-grade Epithelial Dysplasia or Invasive Squamous Cell Carcinoma

**Fig. 2:**
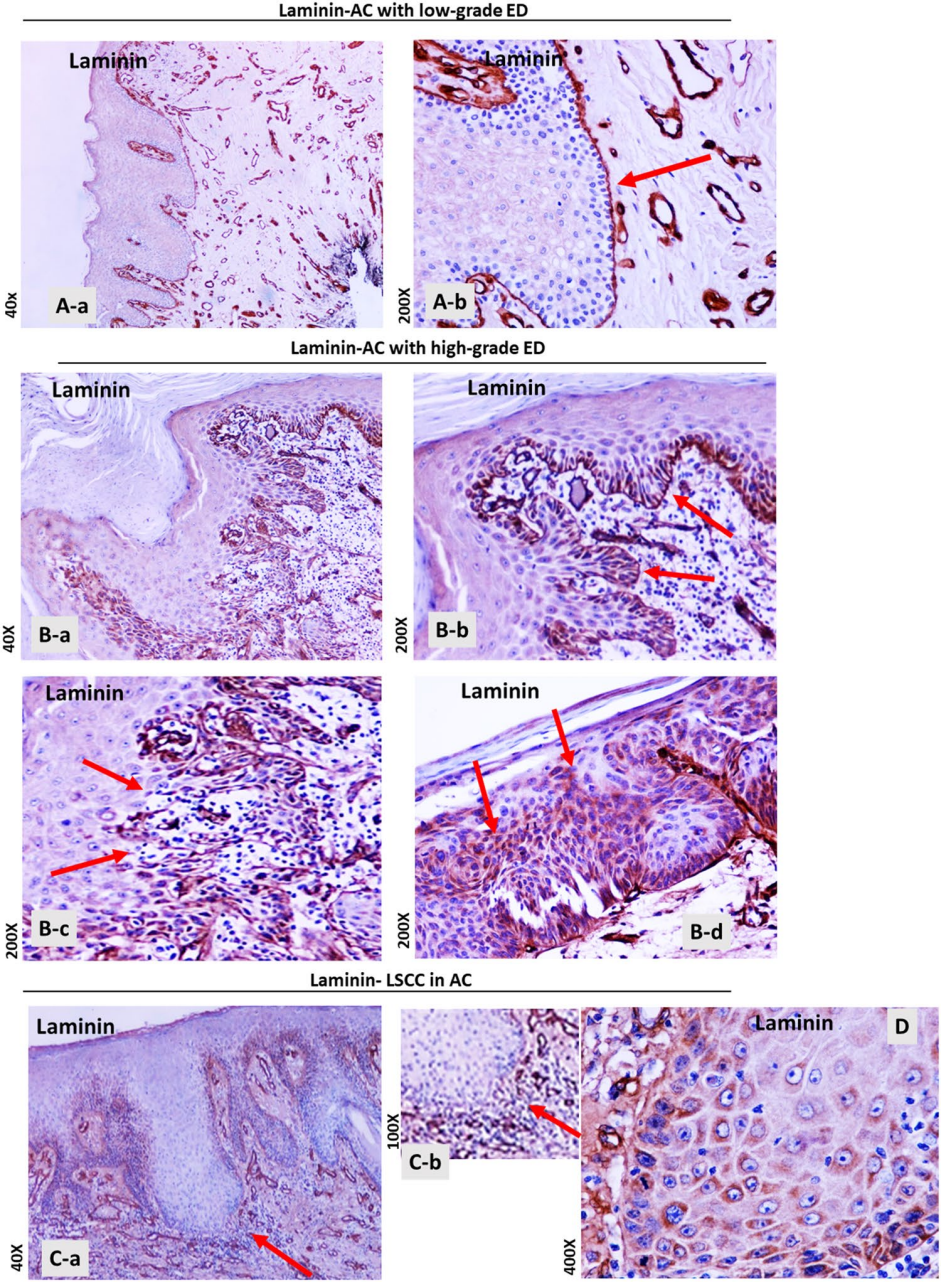
Laminin immunohistochemical (IHC) staining of the basement membrane (BM) in biopsies of actinic cheilitis (AC) with concomitant epithelial dysplasia (ED) and/or invasive squamous cell carcinoma of the lip (LSCC). **A** AC with low-grade ED. Continuous linear staining of the BM with laminin; (a) Original magnification 40x; (b) Original magnification x200. **B** AC with high-grade ED. (a) Original magnification x40; (b) Continuous linear staining of the BM with laminin. Original magnification x200; (c) Focal weak staining of laminin at sites of BM with in *situ* carcinoma. Original magnification x200. (d) Cytoplasmic laminin staining of parabasal cells in high -grade ED of AC. Original magnification x200. **C** AC with squamous cell carcinoma of the lip (LSCC). Original magnification x40. (a) Discontinued laminin staining, indicating fragmentation of the BM. (b) Magnification x100. **D** Cytoplasmic laminin staining in tumor cells. Original magnification x400

IHC evaluation for laminin revealed intense and linear staining of the BM in all AC cases with low-grade ED (Fig. 2 A a,b). Laminin staining also revealed the continuation of BM in AC biopsies with high-grade ED/in situ carcinoma, although it appeared less intense than AC with low-grade ED (Fig. 2B a,b). However, IHC evaluation showed focal and weaker staining of laminin at sites of BM with high-grade ED/in situ carcinoma (Fig. 2B-c). Interestingly, cytoplasmic accumulation of laminin immunostaining was observed in squamous cells of parabasal layers in cases of high-grade ED/in situ carcinoma (Fig. 2B-d).

On the other hand, IHC evaluation for laminin in invasive LSCC revealed loss of its staining in the invasion front, indicating fragmentation of the BM (Fig. 2 C-a,b). Finally, the tumor cells of all the infiltrating LSCC in our study showed cytoplasmic expression of laminin (Fig. 2D).

## Discussion

We present new insights into laminin IHC staining as a diagnostic marker of the progression of AC to invasive LSCC. Specifically, we document the differential staining of laminin in BM between AC with low and high-grade ED or invading LSCC, supporting its use as a histopathologic marker of early neoplastic events and invasion.

The BM is a specialized form of extracellular matrix; its role is to separate the epithelium from underlying connective tissue [43]. Specifically, the BM consists of a mixture of collagens and glycoproteins, such as laminin, that can bind to each other to make a highly cross-linked extracellular matrix [44]. It is known that the integrity of the BM changes during inflammation in order to allow the inflammatory cells enter the epithelium. Most importantly, the BM structure undergoes alterations during neoplastic infiltration of cancer cells [45]. To the best of our knowledge, we present for the first time the differential IHC staining of laminin in AC with ED or LSCC, supporting its role as a useful IHC marker in the differential diagnosis. Specifically, our data showed different patterns of IHC expression of laminin-1 between AC with low-grade ED and AC with high-grade ED/in situ carcinoma and between AC and LSCC developed in AC. According to our findings, diffuse, continuous, and intense laminin staining of the BM is characteristically applied to all biopsies from AC with low-grade ED. Biopsies of AC with high-grade ED/in situ carcinoma also present a continuous expression of laminin, however it may appear less intense, and focal at sites of high-grade/in situ carcinoma than low-grade ED, particularly focally at sites of BM with in *situ* carcinoma. Based on our data, the development of invasive carcinoma in AC demonstrates loss of laminin expression in the BM, as documented in all LSCC biopsies. Our findings are consistent with previous studies by Garcia et al. which showed a differential expression of laminin between oral precancerous and cancerous lesions, supporting a loss of continuity of laminin expression in the epithelial BM from the development of oral carcinomas [35]. Specifically, Garcia et al. noted laminin underexpression along the BM, in 20% of the biopsies with low-grade dysplasia and 57% in high-grade dysplastic lesions, and 70% of oral SCC.

An interesting finding of our study was the cytoplasmic expression of laminin detected in AC with high-grade dysplasia and in LSCC. Specifically, our IHC evaluation for laminin-1, whose expression is largely limited to the epithelial BM, revealed cytoplasmic staining in the lower third of the high-grade ED in AC and in the cytoplasm of infiltrating tumor cells forming focal nests in the dermis. Our findings may support IHC data for the laminin-5 γ2 chain from other studies, which suggested laminin overexpression during the progression of neoplastic disease in invasive oral cancer [34, 37]. Peixoto da-Silva et al. [46] reported negative cytoplasmic staining of the laminin-5 γ2 chain in AC cases, and they also found a cytoplasmic accumulation of laminin-5 in invading cancer cells. Cytoplasmic expression of the γ2 chain of laminin has been previously observed in various types of malignancies, and Peixoto da-Silva et al. suggested it may be due to laminin proteolysis products during neoplastic transformation [46]. Although the exact meaning of this staining remains unknown, the change of laminin expression pattern from linear at the basal cells in low-grade ED to cytoplasmic at the parabasal cells in high-grade ED/in situ carcinoma, not only demonstrates a surrogate biomarker for distinguishing these entities but probably indicates a shift of receptors and cell-cell adhesion molecules from BM to dysplastic epithelial cells, that might contribute to the degradation of the extracellular matrix at the beginning of an invasion. Large-scale studies with different molecules and possibly a combination of methodologies could clarify this phenomenon.

## Conclusion

We suggest that immunochemical analysis of laminin expression can be useful in identifying AC cases suspected of early invasion. Furthermore, we propose that laminin immunostaining may contribute to the differential diagnosis between AC with low and high-grade ED, and between AC and invasive LSCC. Alterations in the staining pattern might provide indirect morphological evidence of BM degradation, indicating malignant transformation. From a clinical point of view, the findings could be particularly useful in selecting patients with a greater tendency to develop squamous cell carcinoma and therefore, providing close follow-up.

## Data Availability

The datasets used and/or analyzed during the current study are available from the corresponding author upon reasonable request.

## References

[1] Aupérin A (2020). Epidemiology of head and neck cancers: an update. Curr Opin Oncol.

[2] Miranda-Filho A, Bray F (2020). Global patterns and trends in cancers of the lip, tongue and mouth. Oral Oncol.

[3] Romagna DV, Oliveira MM, Abreu LG, Stein C, Hugo FN, Teixeira R, Malta DC, Naghavi M, Iser BPM (2022). Incidence and mortality rates of lip, oral cavity, and pharynx cancers in Brazil: time-trend and age-period-cohort analysis from the last 30 years, Global Burden of Disease Study. Rev Soc Bras Med Trop.

[4] Muzaffar J, Bari S, Kirtane K, Chung CH (2021). Recent advances and future directions in clinical management of head and neck squamous cell carcinoma. Cancers.

[5] Antunes JL, Biazevic MG, de Araujo ME, Tomita NE, Chinellato LE, Narvai PC (2001). Trends and spatial distribution of oral cancer mortality in São Paulo, Brazil, 1980–1998. Oral Oncol.

[6] Vartanian JG, Carvalho AL, de Araújo Filho MJ, Junior MH, Magrin J, Kowalski LP (2004). Predictive factors and distribution of lymph node metastasis in lip cancer patients and their implications on the treatment of the neck. Oral Oncol.

[7] Massano J, Regateiro FS, Januário G, Ferreira A (2006). Oral squamous cell carcinoma: review of prognostic and predictive factors. Oral Surg Oral Med Oral Pathol Oral Radiol  Endod.

[8] Parkin DM, Bray F, Ferlay J, Pisani P (2005). Global cancer statistics, 2002. Cancer J Clin.

[9] Neville BW, Day TA (2002). Oral cancer and precancerous lesions. Cancer J Clin.

[10] Du M, Nair R, Jamieson L, Liu Z, Bi P (2020). Incidence Trends of lip, oral cavity, and pharyngeal cancers: global burden of disease 1990–2017. J Dent Res.

[11] Lorini L, Bescós Atín C, Thavaraj S, Müller-Richter U, Alberola Ferranti M, Pamias Romero J, Sáez Barba M, de. Pablo García-Cuenca A, Braña García I, Bossi P, Nuciforo P, Simonetti S, (2021). Overview of oral potentially malignant disorders: from risk factors to specific therapies. Cancers.

[12] Warnakulasuriya S, Ariyawardana A (2016). Malignant transformation of oral leukoplakia: a systematic review of observational studies. J Oral Pathol med.

[13] Wang TY, Chiu YW, Chen YT, Wang YH, Yu HC, Yu CH, Chang YC (2018). Malignant transformation of Taiwanese patients with oral leukoplakia: a nationwide population-based retrospective cohort study. J Formos Med Assoc.

[14] Liu W, Shi LJ, Wu L, Feng JQ, Yang X, Li J, Zhou ZT, Zhang CP (2012). Oral cancer development in patients with leukoplakia–clinicopathological factors affecting outcome. PLoS ONE.

[15] Ayers S (1923). Chronic Actinic Cheilitis. JAMA.

[16] de Santana Sarmento DJ, da Costa Miguel MC, Queiroz LM, Godoy GP, da Silveira EJ (2014). Actinic cheilitis: clinicopathologic profile and association with degree of dysplasia. Int J Dermatol.

[17] Ackerman AB. (2001). Resolving quandaries in dermatology, pathology and dermatopathology. Vol. 2. New York: Ardor Scribendi; 2001a. Actinic cheilitis? p. 20. https://scholar.google.com/scholar_lookup?title=Resolving+quandaries+in+dermatology,+pathology+and+dermatopathology&author=AB+Ackerman&publication_year=2001a&amp.

[18] Warnakulasuriya S (2018). Clinical features and presentation of oral potentially malignant disorders. Oral Surg Oral Med Oral Pathol Oral Radiol.

[19] Cavalcante AS, Anbinder AL, Carvalho YR (2008). Actinic cheilitis: clinical and histological features. J Oral Maxillofac Surg.

[20] Markopoulos A, Albanidou-Farmaki E, Kayavis I (2004). Actinic cheilitis: clinical and pathologic characteristics in 65 cases. Oral Dis.

[21] Vieira RA, Minicucci EM, Marques ME, Marques SA (2012). Actinic cheilitis and squamous cell carcinoma of the lip: clinical, histopathological and immunogenetic aspects. An Bras Dermatol.

[22] de Castro Abrantes T, Fonsêca TC, Cabral MG, Agostini M, Benevenuto de Andrade BA, Romañach MJ, Abrahão AC (2021). Epithelial dysplasia in actinic cheilitis: microscopic study of 70 cases from brazil. Head Neck Pathol.

[23] Dancyger A, Heard V, Huang B, Suley C, Tang D, Ariyawardana A (2018). Malignant transformation of actinic cheilitis: a systematic review of observational studies. J Investig Clin Dent.

[24] Jadotte YT, Schwartz RA (2012). Solar cheilosis: an ominous precursor: part I. Diagnostic insights. J Am Acad Dermatol.

[25] Rass K, Reichrath J (2008). UV damage and DNA repair in malignant melanoma and nonmelanoma skin cancer. Adv Exp Med Biol.

[26] Neto Pimentel DR, Michalany N, Alchorne M, Abreu M, Borra RC, Weckx L (2006). Actinic cheilitis: histopathology and p53. J Cutan Pathol.

[27] Warnakulasuriya S, Kujan O, Aguirre-Urizar JM, Bagan JV, González-Moles M, Kerr AR, Lodi G, Mello FW, Monteiro L, Ogden GR, Sloan P, Johnson NW (2021). Oral potentially malignant disorders: A consensus report from an international seminar on nomenclature and classification, convened by the WHO Collaborating Centre for Oral Cancer. Oral Dis.

[28] Guldager Kring Rasmussen D, Karsadal MA, Karsdal A (2016). Biochemistry of Collagens Laminin and Elastin Structure function and biomarkers. Laminins and Elastin.

[29] Aumailley M (2013). The laminin family. Cell Adh Migr.

[30] Ryan MC, Christiano AM, Engvall E, Wewer UM, Miner JH, Sanes JR, Burgeson RE (1996). The functions of laminins: lessons from in vivo studies. Matrix Biol.

[31] Engbring JA, Kleinman HK (2003). The basement membrane matrix in malignancy. J Pathol.

[32] Yellapurkar S, Natarajan S, Boaz K, Manaktala N, Baliga M, Shetty P, Prasad M, Ravi M (2018). Expression of laminin in oral squamous cell carcinomas. Asian Pac J Cancer Prev.

[33] Firth NA, Reade PC (1996). The prognosis of oral mucosal squamous cell carcinomas: a comparison of clinical and histopathological grading and of laminin and type IV collagen staining. Aust Dent J.

[34] Degen M, Natarajan E, Barron P, Widlund HR, Rheinwald JG (2012). MAPK/ERK-dependent translation factor hyperactivation and dysregulated laminin γ2 expression in oral dysplasia and squamous cell carcinoma. Am J Pathol.

[35] Santos-García A, Abad-Hernández MM, Fonseca-Sánchez E, Julián-González R, Galindo-Villardón P, Cruz-Hernández JJ, Bullón-Sopelana A (2006). E-cadherin, laminin and collagen IV expression in the evolution from dysplasia to oral squamous cell carcinoma. Med Oral Patol Oral Cir Bucal.

[36] Kannan S, Balaram P, Chandran GJ, Pillai MR, Mathew B, Nalinakumari KR, Nair MK (1994). Alterations in expression of basement membrane proteins during tumour progression in oral mucosa. Histopathology.

[37] Rani V, McCullough M, Chandu A (2013). Assessment of laminin-5 in oral dysplasia and squamous cell carcinoma. J Oral Maxillofac Surg.

[38] Barnes L, Eveson JW, Reichart P, Sidransky D, World Health Organization (WHO) (2005). Classification of tumours. Pathology and genetics of head and neck tumours. Blue book, IARC.

[39] El-Naggar AK, Chan JKC, Takata T, Grandis JR, Slootweg PJ (2017). The fourth edition of the head and neck World Health Organization blue book: editors’ perspectives. Hum Pathol.

[40] Ekblom P, Lonai P, Talts JF (2003). Expression and biological role of laminin-1. Matrix Biol.

[41] Vageli DP, Doukas PG, Doukas SG, Tsatsakis A, Judson BL (2022). Noxious combination of tobacco smoke nitrosamines with bile, deoxycholic acid, promotes hypopharyngeal squamous cell carcinoma, via NFκb, in vivo. Cancer Prev Res.

[42] Sasaki CT, Doukas SG, Costa J, Vageli DP (2019). Biliary reflux as a causal factor in hypopharyngeal carcinoma: New clinical evidence and implications. Cancer.

[43] Timpl R (1989). Structure and biological activity of basement membrane proteins. Eur J Biochem.

[44] Ross HM, et al (2006). Histology: A Text and Atlas: With correlated cell and molecular biology (Histology (Ross)): With correlated cell and molecular biology. 5th edition (LWW). Volume 5; pp121-131. https://www.biblio.com/book/histology-text-atlas-correlated-cell-molecular/d/1187438730.

[45] Liotta LA, Tryggvason K, Garbisa S, Hart I, Foltz CM, Shafie S (1980). Metastatic potential correlates with enzymatic degradation of basement membrane collagen. Nature.

[46] Peixoto da-Silva J, Lourenço S, Nico M, Silva FH, Martins MT, Costa-Neves A (2012). Expression of laminin-5 and integrins in actinic cheilitis and superficially invasive squamous cell carcinomas of the lip. Pathol Res Pract.

